# Maternal effects in the model system *Daphnia*: the ecological past meets the epigenetic future

**DOI:** 10.1038/s41437-024-00742-w

**Published:** 2025-01-08

**Authors:** Trenton C. Agrelius, Jeffry L. Dudycha

**Affiliations:** 1 Department of Biological Sciences, University of Notre Dame, Notre Dame, IN USA; 2 Department of Biological Sciences, University of South Carolina, Columbia, SC USA

**Keywords:** Epigenetics, Evolution, Ecology

## Abstract

Maternal effects have been shown to play influential roles in many evolutionary and ecological processes. However, understanding how environmental stimuli induce within-generation responses that transverse across generations remains elusive, particularly when attempting to segregate confounding effects from offspring genotypes. This review synthesizes literature regarding resource- and predation-driven maternal effects in the model system *Daphnia*, detailing how the maternal generation responds to the environmental stimuli and the maternal effects seen in the offspring generation(s). Our goal is to demonstrate the value of *Daphnia* as a model system by showing how general principles of maternal effects emerge from studies on this system. By integrating the results across different types of biotic drivers of maternal effects, we identified broadly applicable shared characteristics: 1. Many, but not all, maternal effects involve offspring size, influencing resistance to starvation, infection, predation, and toxins. 2. Maternal effects manifest more strongly when the offspring’s environment is poor. 3. Strong within-generation responses are typically associated with strong across-generation responses. 4. The timing of the maternal stress matters and can raise or lower the magnitude of the effect on the offspring’s phenotype. 5. Embryonic exposure effects could be mistaken for maternal effects. We outline questions to prioritize for future research and discuss the possibilities for integration of ecologically relevant studies of maternal effects in natural populations with the molecular mechanisms that make them possible, specifically by addressing genetic variation and incorporating information on epigenetics. These small crustaceans can unravel how and why non-genetic information gets passed to future generations.

## Introduction

Maternal effects occur when mothers influence the phenotype of their offspring through means other than genetic inheritance, and their role in a wide range of ecological and evolutionary processes has been extensively studied (Bernardo 1996; Marshall and Uller 2007; Badyaev 2008; Wolf and Wade 2009, 2016; Mousseau et al. 2009; Burgess and Marshall 2014). Two distinct types of maternal effects have been discussed. In maternal genetic effects, the mother’s genotype causally influences the phenotype of the offspring (Wolf and Wade 2009; 2016) In maternal environmental effects, the mother’s environment causally influences the phenotype of the offspring, and as such is a transgenerational form of phenotypic plasticity (Mousseau and Fox 1998; Roach and Wulff 1987; Galloway 2005). Regardless of which type is being considered, most researchers focus on the existence and consequences of maternal effects, rather than the mechanisms of action (but see Grindstaff et al. 2003; Champagne and Curley 2009; Meylan et al. 2012; Groothuis et al. 2019; Venney et al. 2020). Although both types of maternal effects can have substantial consequences for adaptive evolution, here our focus is on maternal environmental effects. For simplicity, we refer to them as maternal effects hereafter.

Maternal effects draw together two important phenomena in evolution: non-genetic inheritance from one generation to the next, and the ability of organisms to produce different phenotypes in response to different environments. Considerable empirical evidence highlights the effects of nongenetic inheritance on a multitude of traits across taxa and generations (reviewed in Bonduriansky et al. 2012; Skinner and Nilsson 2021). Non-genetic inheritance is important because it will influence the rate and direction of adaptation, especially in populations experiencing environmental fluctuations (Jablonka and Lamb 1995; Bernardo 1996; Marshall and Uller 2007; Mousseau et al. 2009; Day and Bonduriansky 2011; Burgess and Marshall 2014). Because maternal effects sometimes appear to reduce offspring fitness, Marshall and Uller (2007) argued that the adaptive value of maternal effects should be considered more broadly than a simple focus on offspring fitness in a single environment. In particular, they advocated for considering how maternal effects influence the mother’s fitness and how context-dependence of multiple offspring phenotypes contribute to effects on offspring fitness. Thus, developing an understanding of the evolutionary ecology of maternal effects requires model systems where multiple generations of ecological information can be combined with reactions norms of phenotypic variation and fitness.

Our understanding of maternal effects is not limited to evolutionary ecology (Galloway et al. 2009; Wolf and Wade 2016; McAdam et al. 2014). Incorporating population-scale genetic variation of the magnitude and direction of maternal effects with an understanding of ecologically realistic fitness consequences allows, in principle, for predicting adaptive evolution. Because maternal effects are potentially governed by a large number of loci, this entails quantitative genetic approaches, and as a consequence, the ability to separate genetic and environmental influences on phenotypes experimentally becomes critical. With maternal effects, this is complicated by the need to further distinguish these between generations.

**Fig. 1:**
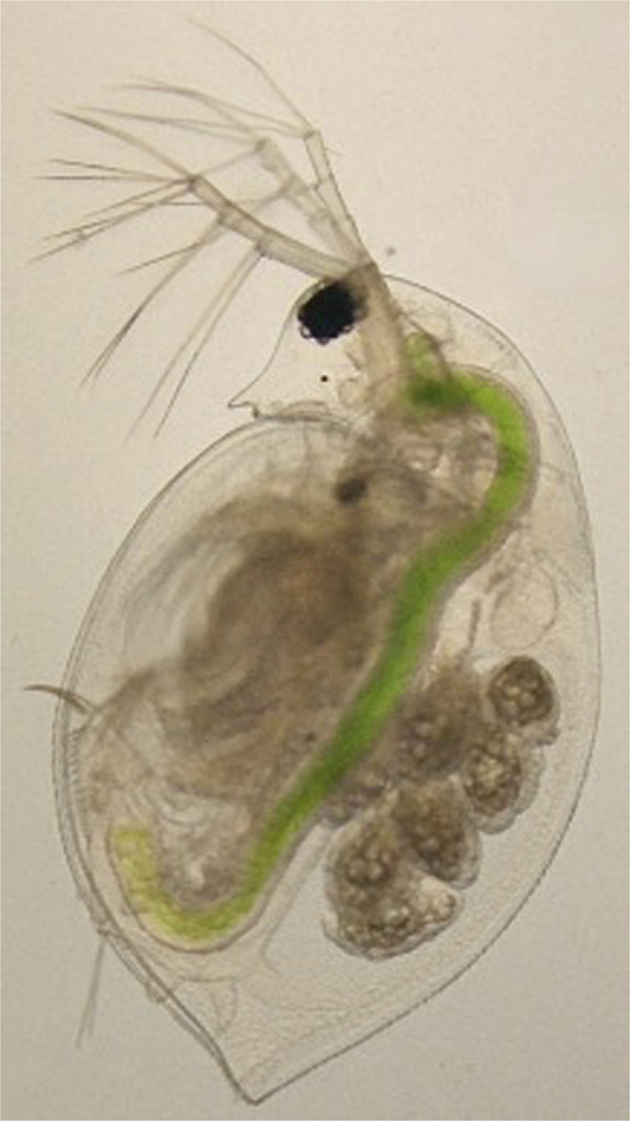
Photograph of an adult female *Daphnia pulex.* *Daphnia* are freshwater, filter feeding microcrustaceans belonging to the superorder Cladocera. The large black circle in the head region is a compound eye. The digestive tract can be easily seen via the bright green color of consumed algae. The multicellular, elongated ovoids within the brood chamber located toward the posterior of the animal are parthenogenetic embryos. Photograph taken by Trenton C. Agrelius with the ventral side to the left and head is oriented to the top.

While the combination of evolutionary ecology and quantitative genetics can provide a powerful insight into the evolutionary dynamics of maternal effects, physiology, and molecular biology are needed to understand the mechanisms of action by which maternal effects occur. Such mechanistic knowledge creates the potential to investigate the evolution of the control systems regulating maternal effects. Thus, an integrated understanding of the evolution of maternal effects will draw on ecology, quantitative genetics, physiology, and molecular biology. The freshwater crustacean *Daphnia* (Fig. 1) is a model system for studying maternal effects in which these four fields of biology can readily be integrated.

**Fig. 2:**
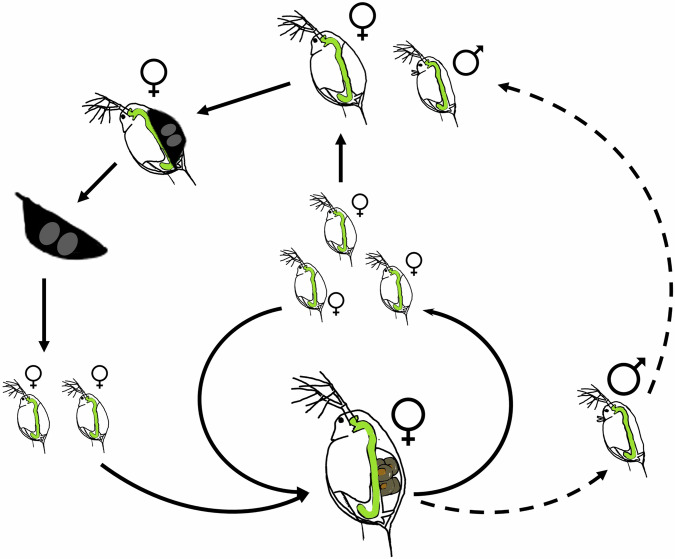
Diagram of the cyclically parthenogenetic life cycle common to most *Daphnia.* In the field, multiple parthenogenetic (asexual) generations usually occur before a single generation of sexual reproduction. Reproductive modes seasonally switch in response to environmental changes, such as temperature changes or food scarcity. Parthenogenetically produced eggs develop immediately and are carried in the brood chamber until their release as neonates. In contrast, sexual reproduction involves fertilized eggs that are encapsulated in a structure known as an ephippium and enter dormancy. Under laboratory conditions, clonal lines can be maintained through continuous asexual reproduction.

*Daphnia* are a classic model in research on phenotypic plasticity, and indeed is the system where Woltereck (1909) originally developed the concept of reaction norms. One of the major advantages of *Daphnia* is their cyclically parthenogenetic life cycle (Fig. 2). Parthenogenetically produced diploid eggs develop immediately (Zaffagnini 1987) and result in genetically identical offspring, barring mutation. This allows researchers to expose clones to different environments, easily determining the environmental drivers of phenotypic variation. However, the sexual phase of the life cycles means that natural (meta)populations contain substantial genetic diversity (e.g., Pfrender and Lynch 2000; Haag et al. 2005; Walser and Haag 2012; Lynch et al. 2017), and thus genetic variation of the capacity for phenotypic plasticity can also be investigated (e.g., De Meester 1996; Weber and Declerck 1997; Scheiner and Berrigan 1998; Landy et al. 2020; Becker et al. 2022). The asexual reproduction also allows for relatively simple analyses of transgenerational phenotypic plasticity, since researchers do not need to account for genetic differences between mother and offspring and responses to an environmental cue in the maternal generation can be unambiguously determined (Walsh et al. 2014). While this is possible with recombinant inbred lines in some genetic model organisms, such explorations may suffer from variable effects of inbreeding depression. Artificially homozygous genomes may also poorly reflect individuals of natural populations. However, asexual reproduction in *Daphnia* also largely precludes investigations into maternal genetic effects.

A second major advantage of using *Daphnia* in studies of maternal effects is the wealth of ecological information on them (reviewed in Ebert 2022). *Daphnia* are keystone species in freshwater food webs, and the ease of field observations and experimental manipulations in the lab and field have made them the genus whose ecology is perhaps the best known. This base of information means that the natural context and consequences of virtually any significant ecological axis has been established for at least some species. Thus, experimental conditions for investigations into maternal effects can be chosen that reflect real ecology. In particular, the scale of likely environmental variation between generations is important for understanding how maternal effects influence evolution. In many cases, physiological ecologists have established the mechanisms by which environmental variation produces phenotypic and other ecological outcomes with *Daphnia* (e.g., Lee 1984; Pinkhaus et al. 2007; Dennis et al. 2014; Yampolsky et al. 2014; Weiss et al. 2015a, 2015b).

Finally, *Daphnia* are an advantageous model system due to recent advances in genomics and functional molecular biology, largely driven by researchers motivated by evolutionary questions (Colbourne et al. 2011; Miner et al. 2012; Ebert 2022). This research has generated an extensive body of knowledge on molecular genetic variation in a few species (Lynch et al. 2017; Fields et al. 2022). Functional molecular biology has included establishing some understanding of gene expression, much of which is ecologically grounded (Decaestecker et al. 2011; Dudycha et al. 2012; Orsini et al. 2016; Hales et al. 2017). In addition, epigenetics, a domain of molecular biology with particular relevance for maternal effects (Youngson and Whitelaw 2008; Anastasiadi et al. 2021; Das et al. 2022), is beginning to be studied in *Daphnia*. Researchers have also developed manipulative genetic tools, including RNAi (Kato et al. 2011; Schumpert et al. 2015) and CRISPR (Kumagai et al. 2017; Ismail et al. 2018), that allow for research into molecular mechanisms. Together, these advances will allow integrating research on molecular mechanisms of maternal effects with our understanding of their ecology and evolution.

Many reviews of maternal effects have been written (e.g., Mousseau and Fox 1998; Räsänen and Kruuk 2007; Wolf and Wade 2009; Heard and Martienssen 2014), but some have ignored *Daphnia*, and none have focused on it. We believe a review of *Daphnia* is warranted due to its unique potential for integrating ecological, evolutionary, and mechanistic aspects of maternal effects. This review synthesizes literature regarding maternal effects induced by the resource and predation environments experienced by *Daphnia*. We exclude the extensive research on maternal effects linked to temperature and photoperiod (e.g., Betini et al. 2020) to keep the review manageable. Our goals here are threefold. We argue for the importance of *Daphnia* as a model system by presenting how their attributes have led them to become one of the richest model systems in maternal effects. Second, we synthesize the results across different types of drivers of maternal effects to identify shared patterns that may reflect broad principles of maternal effects, despite highlighting some notable exceptions. Finally, we discuss the possibilities for future integration of ecologically relevant studies of maternal effects in natural populations with the molecular mechanisms that make them possible. In particular, we argue for the need for future work to address genetic variation of the sensitivity and magnitude of maternal effects, and to incorporate information on the action of epigenetics and maternal provisioning.

## Diet: what quantity and quality can do for you

### Part I. Resource quantity (mgC/L; number of cells)

*Daphnia* respond to food availability by adjusting phenotypes within and across generations. In response to low resources, *Daphnia* exhibit many life history changes including reduced body size, smaller clutches, slower growth, delayed maturation, and extended lifespan (Lynch 1989; Weider 1993; Dudycha and Lynch 2005). At an organism-scale, functional systems also respond to low ambient resources. In response to low resource availability, feeding physiology and behavior (Lampert 1994; Lampert and Brendelberger 1996), and investment in sensory systems decreases (Brandon and Dudycha 2014). Recent studies have explored resource responses at a molecular level, revealing differential gene expression and altered methylation of over 100 genes under resource limitation (Hearn et al. 2019). Additionally, under resource limitation, epigenetic modifiers like DNA methyltransferases are upregulated (Nguyen et al. 2020; Agrelius et al. 2023).

Manipulative experiments controlling food level show positive correlations across resource levels between maternal body size and clutch size and between maternal body size and offspring size, with all of these traits increasing with resource level (e.g., Glaizer 1991; Gliwicz and Guisande 1992; Mckee and Ebert 1996). These traits have correlations among themselves that may in part be driven by maternal effects which may also be driven by the structure of life history tradeoffs. Nonetheless, offspring size-number tradeoffs still produce a negative correlation between offspring size and maternal clutch size (Gabsi et al. 2014), with differences in the quality, number, and size of the neonates produced, as detailed below.

Maternal provisioning of eggs dynamically responds to resource limitation, with a shift from producing more eggs to fewer eggs of greater size and elemental content. *Daphnia* reared in low-resource environments produce larger eggs with higher carbon, nitrogen, protein, and lipid content than high resource environments (Boersma 1997; Guisande and Gliwicz 1992). This trend of larger but fewer, well-provisioned eggs under low food availability is well-documented in *D. magna* (Glaizer 1991; Boersma 1997; Burns 1995; Pieters et al. 2005; Mckee and Ebert 1996; Gorbi et al. 2011; Gabsi et al. 2014; Garbutt and Little 2014; Coakley et al. 2018; Hearn et al. 2018), *D. pulicaria* (Tessier and Consolatti 1989; Gliwicz and Guisande 1992)*, D. pulex* (Taylor 1985; Tessier and Consolatti 1989; Li and Jiang 2014)*, D. hyalina* (Gliwicz and Guisande 1992)*, D. galeata, D. mendotate*, and *D. parvula* (Tessier and Consolatti 1989). More recent data suggest that *Daphnia* also provision eggs with microRNAs that match the maternal differential gene expression pattern when mothers are reared in low resource environments (Hearn et al. 2018).

Maternal resource limitation also influences embryogenesis, in some cases prolonging embryonic development (Guisande and Gliwicz 1992). Embryonic development may be an important mediator of maternal effects by altering an individual’s starting point on the fast-slow continuum of life history, but not much attention has been given to the correlation between the plasticity of embryogenesis and offspring life history. Hasoon and Plaistow (2020) showed a consistent association between embryonic developmental stage durations and offspring life history traits in *D. pulex*, with egg length and duration of specific developmental stages correlating with offspring size, time to maturity, and fecundity for the first three clutches (Hasoon and Plaistow 2020). For instance, larger eggs were reported to have longer embryonic development times for stages 3, 4, and 8 but shorter developmental times in stages 2 and 5, correlating with delayed maturation at a larger size and production of larger offspring (Hasoon and Plaistow 2020). In some cases, the durations of embryonic developmental stages were better correlated with offspring traits than the more commonly reported metric of egg size. *Daphnia* eggs have differential miRNA expression when the maternal generation experiences either food stress or increased age (Hearn et al. 2018) indicating that embryonic development is plastic and responsive to maternal cues. This plasticity likely contributes to the variation seen in offspring life history.

Offspring from *Daphnia* reared in low resource environments are generally larger at birth, which correlates with improved starvation resistance, decreased age at first maturation, and increased fecundity (Porter et al. 1983; Tillmann and Lampert 1984; Lampert 1993), than *Daphnia* reared in high resource environments. These offspring exhibit lower feeding rates (Garbutt and Little 2014) and significantly lower carbon loss during development (Boersma 1995) while performing better in environments similar to those experienced by their mothers. The impact of maternal environment on offspring phenotype can vary across species and clones (Glazier 1992; Guisande and Gliwicz 1992; Gorbi et al. 2011). In one experiment, *D. pulex* responded to low ambient resources via a type of generational memory in which a mismatch between maternal and offspring environments resulted in offspring switching to producing resting eggs (LaMontagne and McCauley 2001). Tessier and Consolatti (1991) observed that *D. pulicaria’s* response to low and high resource environments was qualitatively similar to *D. pulex* clones used in their study, but the magnitude of the response significantly varied between the two species.

The pattern of producing larger offspring under food limitation is also dependent on the degree of limitation and the quality of the resource. Glazier (1992) proposed a model accounting for maternal energetic demand where the largest offspring would be produced under “intermediate” food levels, a categorization that is dependent upon the quality of food source used (Taylor 1985; McKee and Ebert 1996). Most resource availability studies rely on either the carbon content (mgC/L) or the number of cells used for feeding regimes. Comparing resource quantity studies, especially concerning maternal effects, becomes challenging due to different standards used to define low resource quantity. Moreover, feeding *Daphnia* abundant low-quality resources can yield responses similar to those of low-resource diets and vice versa.

### Part II. Resource quality (nutrient and elemental compositions)

Dietary nutrition in *Daphnia* encompasses the elemental and biochemical compositions of consumed algal species, as well as their physical shape, size, and digestibility (Becker and Boersma 2003; Ilic et al. 2019; DeMott 1990). Many studies focus on carbon-to-nutrient ratios (C:P, C:N) (e.g., Sterner and Elser 2002; Frost et al. 2010) or the fatty acid content of phytoplankton (e.g., Stoecker and Capuzzo 1990; Martin-Creuzburg et al. 2008). While absolute carbon content is typically considered a measure resource quantity (mgC/L), the levels of elements such as phosphorus and nitrogen within phytoplankton strongly affect *Daphnia* fitness.

Diets with low nutrient content or relatively high carbon:nutrient ratios are considered low quality. Reproduction is likely a phosphorus sink, as offspring exhibit higher phosphorus content than their mothers even in high-phosphorus environments (Frost et al. 2010). *Daphnia* reared in phosphorus-limited conditions display lower mass-specific phosphorus content, leading to delayed maturation, smaller clutch sizes, and increased mortality (DeMott et al. 1998; Boersma and Kreutzer 2002; Sterner and Elser 2002; Frost et al. 2010). Phosphorus limitation also prompts *Daphnia* mothers to increase phosphorus and sterol provisioning in offspring compared to those reared in high-phosphorus conditions (Boersma and Kreutzer 2002; DeMott et al. 1998).

Maternal phosphorus limitation results in offspring with reduced mass and phosphorus content (DeMott et al. 1998; Urabe and Sterner 2001) and smaller body size (Frost et al. 2010). Frost et al. (2010) induced phosphorus limitation by increasing the C:P ratio and observed that maternal phosphorus limitation was transferred to offspring, leading to reduced juvenile-specific growth rate, delayed maturation, and potentially increased susceptibility to *Pasteuria ramosa* infection. The effects of maternal diet were observed to be strongest when the daughters’ resource quality was also phosphorus limited. When offspring from mothers reared under high C:P conditions were grown in phosphorus-rich media with low C:P ratios, the negative effects of maternal phosphorus stress on growth rate and reproduction in juveniles were nearly eliminated. These findings demonstrate a transgenerational transfer of stress induced by maternal diet quality, with the strength of the stress varying based on offspring diet quality.

In general, when an offspring consumes a low-quality or poor-quality diet, the effect size and magnitude of the mother’s diet on the offspring’s life history becomes paramount. The strength of the maternal effect is greatest and most pronounced in early development stages and gradually weakens over time (Brett 1993; Hearn et al. 2018), influencing embryonic development, age and size at maturation, clutch size, and egg size. Differentially expressed miRNAs were observed in nutritionally stressed *Daphnia* and their eggs (Hearn et al. 2018), but miRNA expression was not maintained into offspring adulthood and subsequently lost in the grandmaternal generation. Conversely, when the offspring resource base is high and good quality, maternal effects on offspring fitness are at most transitory and often unseen because offspring have access to quality resources necessary for normal developmental trajectories. Maternal effects may partially compensate for poor offspring environments, but the effect size of direct environmental exposure on the offspring will generally be greater than maternal influence.

### Biochemical diet: sterols

*Daphnia* rely on external sources of both highly unsaturated fatty acids (HUFA) and polyunsaturated fatty acids (PUFA) (Stoecker and Capuzzo 1990). *Daphnia* lack the ability to synthesize PUFAs de novo (Stanley-Samuelson 1987; Leonard et al. 2004), but there is evidence of PUFA bioconversion in some species (Weers et al. 1997; Kainz et al. 2004; Schlechtriem et al. 2006; Burns et al. 2011). Studies investigating HUFA and PUFA deficiencies have yielded diverse results, including impaired membrane and enzymatic system function leading to reduced growth, decreased fecundity (Stoecker and Capuzzo 1990; Sperfeld and Wacker 2015; Ilic et al. 2019), increased disease susceptibility (Schlotz et al. 2013), enhanced starvation resistance (Becker and Boersma 2007), and altered phototaxis behavior (Michels and DeMeester 1998).

Data clearly show that the fatty acid composition of *Daphnia* is quantitatively similar to their diets (Müller-Navarra 2006; Wacker and Martin-Creuzburg 2007; Sperfeld and Wacker 2012; Schlotz et al. 2013), and that *Daphnia* mothers are capable of regulating the amount and type of sterol allocated to eggs (Wacker and Martin-Creuzburg 2007; Sperfeld and Wacker 2015). Sperfeld and Wacker (2015) showed that the PUFA profiles of eggs and offspring correspond quantitatively to the fatty acid composition of the maternal diet. However, maternal allocation of fatty acids into eggs and offspring tissue increased when the maternal diet consisted of low-quality diets with relatively low PUFA content. Nevertheless, the literature is inconsistent regarding the specific importance of certain fatty acids and their effects on *Daphnia* spp. (von Elert 2002; Martin-Creuzburg et al. 2010; Ravet et al. 2012; Schlotz et al. 2014; Ilic et al. 2019).

Maternal effects resulting from sterol provision impact several fitness parameters including juvenile specific growth rate, infection resistance, total reproductive output, and starvation resistance. Juvenile specific growth rate of *Daphnia* is commonly accepted as a proxy for fitness (Lampert and Trubetskova 1996), and there is strong evidence that egg PUFA content correlates with juvenile specific growth rate, particularly eicosapentaenoic (Müller-Navarra 1995) and alpha-linolenic acid (Wacker and von Elert 2001; Sperfeld and Wacker 2015; Ilic et al. 2019). Both acids are substrates for eicosanoids, a family of hormone-like substances that act on reproduction, ion transport physiology, and the immune system (Stanley 2000). Schlotz et al. (2013) found that diets rich in PUFAs resulted in increased infection resistance for the maternal generation but a six-fold increase in infection for offspring. Schlotz et al. (2013) also found that maternal diets rich in PUFAs resulted in offspring producing the same number of offspring as their mothers over a period of 30 days despite never having consumed a PUFA rich diet. Finally, data from Becker and Boersma (2007) show that offspring from mothers that were provisioned with high levels of PUFAs were more resistant to starvation and had a positive growth rate when subjected to a low resource environment.

### Elemental diet: biotic-lite, calcium

Most research on resource-driven maternal effects in *Daphnia* focuses on diet, but not all resources are obtained this way. Cowgill et al. (1986) demonstrated that calcium (Ca) is primarily acquired from the environment through readily available dissolved sources, rather than from dietary intake. Calcium plays a crucial role in the *Daphnia* life cycle, and there is evidence that it influences maternal effects through provisioning.

During each molt, *Daphnia* lose ~90% of their body calcium (Alstad et al. 1999). To compensate for this loss, *Daphnia* actively take up large amounts of calcium from the water. *Daphnia* lack the ability to store calcium for an extended period, and their capacity to prolong the calcification process, which begins shortly before molting, is limited (Porcella et al. 1969, reviewed in Cairns and Yan 2009). The specific calcium requirements vary depending on the developmental stage (Hessen et al. 2000) and the consequences of calcium limitation differ based on when it is experienced (Cairns and Yan 2009). Osmoregulatory organs responsible for calcium transfer develop after embryos hatch from the vitelline membrane but before they are released from the brood chamber (Charmantier and Charmantier-Daures 2001). Generally, female *Daphnia* that are calcium-limited exhibit smaller size and delayed maturation but produce a greater number of offspring compared to those reared in high-calcium conditions (Cairns and Yan 2009, references within; Giardini et al. 2015). Juvenile *Daphnia* and other cladocera require higher amounts of calcium per body mass relative to adults and have a faster mineral uptake rate (Tan and Wang 2009).

Giardini et al. (2015) traced radiolabeled calcium from mothers to offspring, suggesting that *Daphnia* are capable of actively regulating the provisioning of calcium to embryos. However, maternal provisions seem to be depleted before osmoregulatory organs have fully formed in the embryo (Giardini et al. 2015). No evidence of radiolabeled-Ca uptake within the first 48-h of development was found, and embryos extracted from mothers and placed in zero-calcium environments experienced a significant increase in mortality. Furthermore, calcium concentration in the maternal environment influenced embryonic development rate. Embryos from mothers reared in a low-calcium environment completed embryonic development 5 h earlier than embryos from mothers reared in a high-calcium environment -- without incurring physical abnormalities.

Significant differences were also observed across generations. Giardini et al. (2015) reported that *D. magna* females reared in high calcium increased body size but decreased the number of offspring produced in the first generation. In the second generation, effects on body size were diminished slightly. The number of offspring produced relative to the first generation increased in high Ca, but decreased in low Ca, though there was no difference between the Ca environments in the number of offspring produced by the second generation.

## Toxic algae: when food fights back

Researchers have used both laboratory (e.g., Lampert 1981, 1987; DeMott et al. 1991; Reinikainen et al. 1994, 1995; Ortiz-Rodríguez et al. 2012) and field (e.g., Haney 1987; Sarnelle 1993) experiments to investigate the effects of toxic cyanobacteria on *Daphnia*. Most commonly, this work focuses on *Microcystis aeruginosa* and the hepatotoxin microcystin. Microcystin exposure leads to a reduction in survival, body size, and number of offspring produced (e.g., Arnold 1971; Glazier 1992; Reinikainen et al. 1994, 1995; Rohrlack et al. 1999; Trubetskova and Haney 2006; Schwarzenberger et al. 2010; Schwarzenberger and Von Elert 2013). However, *Daphnia* continuously exposed to *Microcystis* increase their clutch size over time indicating further plasticity that allows for population-scale toxin tolerance (Gustafsson et al. 2005). Furthermore, recovery from toxin-induced effects is possible following the removal of *Microcystis* from the diet (Brett 1993). The within-genaration life history plasticity may be a consequence of microcystin inhibiting digestive enzymes and/or other molecular effects (Schwarzenberger et al. 2010; Asselman et al. 2012; von Elert et al. 2012). *Daphnia* can also achieve tolerance within a generation by remodeling a digestive chymotrypsin (von Elert et al. 2012) and increasing expression of trypsin (Schwarzenberger and Von Elert 2013).

Maternal effects induced by exposure to *Microcystis* are strongest when the exposure continues into the offspring generation (Gustafsson et al. 2005; Ortiz-Rodríguez et al. 2012; Schwarzenberger and Von Elert 2013). Even though *Microcystis* reduces fecundity in the maternal generation, Gustafsson et al. (2005) showed in a single clone of *D. magna* that offspring of exposed mothers inherit some form of protection. These protected offspring went on to have ~30% higher fecundity in a *Microcystis* environment than the unprotected offspring of unexposed mothers. Unprotected offspring delayed maturation by 1.5 days relative to protected offspring, and further delayed each subsequent clutch. Together, these effects resulted in a 28% higher intrinsic rate of population increase (*r*) for protected offspring than unprotected offspring. They also tested for grandmaternal effects and found no evidence to support them.

Maternal effects of *Microcystis* are not uniform across species or clones. Jiang et al. (2014) assayed three clones of *D. carinata* in which only one clone showed a maternal effect of *Microcystis*. *D. magna* and *D. carinata* have similar population growth rates, but *D. magna* accelerated life history in offspring of mothers exposed to *Microcystis* (Gustafsson et al. 2005), while lifespans were extended in *D. carinata* (Jiang et al. 2014). Interestingly, the ability of offspring to switch to a more toxin-tolerant form of chymotrypsin is even seen in species that do not co-exist with cyanobacteria (von Elert et al. 2012), suggesting a universal response within all *Daphnia*. *D. pulicaria* exposed to non-toxic cyanobacteria showed clear shifts in life-history and reduced fitness but no significant maternal effects on average (Gills and Walsh 2019). However, the authors argued that genetic variation among the forty-five clones in their study could have obscured any maternal effects, for example if effects varied qualitatively among clones (Box 1).

When exposed to microcystins, *D. magna* offspring from mothers who themselves had been exposed to microcystins for 1 week had a higher survival rate than do offspring from unexposed or briefly exposed mothers (Ortiz-Rodríguez et al. 2012). They argued this is potentially due to more efficient oxidative protection, increased metabolic rates, and detoxification of microcystins, all of which were inferred from changes in the enzymatic activity of catalase, malate dehydrogenase (MD), and glutathione S transferase (GST), respectively. The increased expression of GST and MD seen in the offspring generation was directly correlated with the duration of time the mother was exposed (Ortiz-Rodríguez et al. 2012). One-week maternal exposure to microcystin elicited increased catalase activity in offspring that correlated with increased survival compared to offspring from unexposed mothers. Separate work has shown that higher levels of trypsin were detected in neonates from mothers exposed to *Microcystis* compared to neonates from unexposed mothers, even when exposure was to non-toxic strains of *Microcystis* (Schwarzenberger and Von Elert 2013).

Box 1 Definitions of key terms in this review **Physiology**. The manner in which a living individual and its parts function.

**Phenotypic plasticity**. The capacity of an individual (or genome, or gene) to express multiple phenotypes, usually in response to environmental variation.

**Maternal effects**. The alteration of offspring phenotypes by mothers irrespective of offspring genotypes.

**Genetic inheritance**. Information transmission from parent to offspring via DNA sequence.

**Non-genetic inheritance**. Information transmission from parent to offspring via mechanisms other than the DNA sequence.

**Maternal provisioning**. Those elements, other than DNA itself, with which a mother endows her offspring. Maternal provisioning can include energy, nutrients, macromolecules (e.g., RNA molecules, antibodies), and information.

**Transgenerational phenotypic plasticity**. Phenotypic plasticity that occurs across generations in response to environmental variation in a prior generation.

**Epigenetics**. Molecular mechanisms that produce control development, physiology, or phenotypes by regulating gene expression rather than changing the sequence of DNA. Some epigenetic mechanisms can involve transmission from parent to offspring.

## Predation: when under attack, does mother know best?

*Daphnia* are exceptionally great candidates for phenotypic plasticity studies exploring the effects of predators on their prey. Upon detection of chemical cues released by predators, kairomones, *Daphnia* can alter their life history traits as well as develop exaggerated morphological features (Krueger and Dodson 1981; Havel and Dodson 1984; Tollrian 1995; Walls et al. 1997; Agrawal et al. 1999; Dzialowski et al. 2003; Imai et al. 2009; Graeve et al. 2021). There is also evidence for strong maternal effects in response to both vertebrate and invertebrate predation capable of lasting for at least two generations (Agrawal et al. 1999; Walsh et al. 2015).

Predation by *Chaoborus, Notonecta*, and planktivorous fish are well documented (Tollrian 1995; reviewed and meta-analysis Riessen 1999; Weiss et al. 2012; Barbosa et al. 2014) and show clear differences in how *Daphnia* respond to different predators. Exposure to *Chaoborus* signals for some *Daphnia* species (e.g*., D. pulex*) to increase body size, delaying maturation and reproducing at a larger size (Hebert and Grewe 1985; Tollrian 1995; Walls et al. 1997; Imai et al. 2009). Vitellogenin gene expression, the major yolk protein precursor (Zaffagnini 1987; Kato et al. 2004) is increased (Rozenberg et al. 2015), presumably leading to the increase in offspring size following maternal exposure to *Chaoborus* observed by Tollrian (1995). Similar responses are seen with *Notonecta* and *D. carinata* or *D. longicephala*, respectively (Grant and Bayly 1981; Dodson 1988a; Weiss et al. 2015a); however, evidence regarding responses to notonectids is quite variable (Riessen 1999). Conversely, fish predation exposure typically results in earlier maturation with a smaller body size, increased clutch size (Dodson 1988a; Tollrian 1994), and the adoption of vertical migration patterns (Dodson 1988b; De Meester 1993). There is evidence that the effects induced by predation on body size are dependent on which instar the kairomone is detected rather than continuous presence of kairomone in the water (Imai et al. 2009; Mikulski and Pijanowska 2010; Miyakawa et al. 2010).

Maternal exposure to kairomones from *Chaoborus* determines the size and number of embryos produced in future generations but does not induce neckteeth formation in *D. pulex* (Imai et al. 2009). Offspring from mothers exposed to *Chaoborus* kairomones produce fewer but larger eggs after delaying maturation (Tollrian 1995). Direct exposure during embryonic development is reported to either produce neonates with strong polyphenisms (Imai et al. 2009, e.g., *D. pulex* neckteeth) or control different life history parameters (Mikulski and Pijanowska 2017, e.g., *D. magna*) when compared to animals relying on maternal exposure only. Mikulski and Pijanowska (2017) report direct embryonic exposure-controlled life history traits like the size and time of neonate release while maternal exposure, or the interaction of maternal and direct exposure, mediated egg holding times, number of neonates produced, size and age at first reproduction. To ensure that maternal exposure was the only influence, Mikulski and Pijanowska (2010; 2017) limited kairomone exposure up to specific instar stages or up to vitellogenesis, respectively. Imai et al. (2009) reports kairomone exposure during the embryonic development of some clutches but discarded animals that would have been exposed as an embryo. Dzialowski et al. (2003) showed that kairomone exposure as juveniles did not induce the long spines characteristic of *D. lumholtzi’*s defensive response. Together, these suggest that the embryonic period is a critical time for strong induction of alternate morphs in some *Daphnia* species.

Maternal exposure to kairomones from fish or *Notonecta* predation shape life history traits in offspring by decreasing offspring size at maturation and the duration of egg holding time in the brood chamber, while increasing offspring clutch sizes (Stibor and Lmapert 2000; Mikulski and Pijanowska 2010, 2017; Walsh et al. 2015, 2016) compared to offspring from naïve mothers. The strength of the maternal effect increases when the mother is exposed to kairomones in her fourth post-embryonic instar. Mothers who show the greatest change in their own phenotype also have the strongest influence on offspring phenotypes (Mikulski and Pijanowska 2010), an observation that contrasts with results from studies using *D*. *ambigua* detailed below.

Timing of the exposure and the difference between maternal vs embryonic perception of the threat can determine the phenotypic outcome for several generations. Kairomones induce a complex suite of traits that include the neuronal and endocrine pathways as well as the expression of morphogenetic factors (Barry 2002; Miyakawa et al. 2010; Weiss et al. 2012; Weiss et al. 2015b; reviewed in Weiss 2019) and vitellogenin genes (Rozenberg et al. 2015). Embryos of *D. pulex* experience a kairomone-sensitive period for neckteeth induction that starts at the third embryonic stage and may persist through the third post-natal instar (Imai et al. 2009); Naraki et al. (2013) and Weiss et al. (2016) report a somewhat narrower sensitive period, In contrast, Miyakawa et al. (2010) reports kairomone reception up to the seventh post-natal instar. Experiments attempting to parse maternal effects from direct embryonic exposure carefully control for this by removing the mother from media containing kairomones once vitellogenesis (darkening of ovaries) occurs and preventing embryonic detection of the kairomone.

### *Daphnia* ambigua: the little clone that defies theory

An intriguing finding to come out of *Daphnia* predator studies is the clone-specific, negative correlation between within- and across-generation plasticity found by Walsh et al. (2015, 2016) in *D. ambigua*. A large-scale analysis of the effects of genotype on plasticity revealed a negative trend in which *D. ambigua* clones either showed strong within-generation plasticity, where females modified their own morphology and or life history traits, or strong across-generation plasticity (maternal effect) that altered offspring phenotype.

The across-generation (maternal) effect lasted for two generations post-removal of the predator cue and was similar in magnitude to that when *Daphnia* were exposed to injured conspecifics. Theory predicted that if conditions remained similar, or were predictable across generations, that there would be increased plasticity both within- and across generations (e.g., Herrera and Bazaga 2010). Maternal effects in this context should be altering offspring in the same direction and increasing in magnitude at the same rate as the as within-generation plasticity, but that was not observed in *D. ambigua* clones that lived under constant fish predation (*Aloas pseudoharengus*). These clones mature earlier when exposed to the kairomone but program their offspring to delay maturation by adding extra instars to their development and decreasing average clutch size. In contrast, clones from lakes in which fish predation was either nonexistent or temporary delayed maturation in the maternal generation but programmed offspring to mature earlier with a faster development rate, resulting in a higher intrinsic rate of population increase, *r*.

Gene expression analysis of one *D. ambigua* clone known to exhibit strong across- but weak within-generation plasticity, revealed upregulation of many sets of genes that are linked with phenotypic responses corelated to fish predation (Hales et al. 2017 and references within). Initial exposure saw differential expression of ~50 genes related to reproductive efforts in the maternal generation while the offspring and grand offspring increased the number of differentially expressed genes, to 223 and 170 respectively, with 121 genes overlapping. Most differentially expressed genes were related to components of the exoskeleton or ribosome activity.

Given that no predator cue was used after maternal exposure, Hales et al. (2017) argued that the decay of differentially expressed genes from the daughter to granddaughter generation suggests potential epigenetic programming. Shifts in DNA methylation for 2002 genes were previously observed using the same *D. ambigua* clone between offspring and grand offspring following exposure to fish kairomones (Schield et al. 2016). Together, this work suggests that *Daphnia* that employ a plastic approach to predation with a strong transgenerational effect plausibly use epigenetic mechanisms to program instructions for their offspring to alter phenotypic traits.

## Discussion

*Daphnia* are well-known to respond phenotypically within generations to a variety of environmental stimuli, but they are also able to extend phenotypic responses across generations. Life history changes that prioritize producing larger offspring, packaged with altered provisioning and carrying modified epigenetic coding, indicate that many across-generation maternal effects will influence major fitness components. The variation of responses seen in *Daphnia* provide opportunities to understand interactions among different processes. Synthesizing the information from the studies reported here, several general conclusions emerge:

1. Many, but not all, maternal effects involve alteration of offspring size. When offspring size is relatively large, that generation is more resistant to starvation, infection, most invertebrate predation, and toxins.
2. Maternal effects manifest more strongly when the offspring’s own environment is poor, particularly with respect to diet.
3. Strong within-generation responses are typically associated with strong across-generation responses. The response of *D. ambigua* to fish predation is an exception.
4. The timing of the maternal stress matters and can raise or lower the magnitude of the effect on the offspring’s phenotype.
5. Embryonic exposure effects can be mistaken for maternal effects. Once active transport and sensory mechanisms have developed in the embryo, distinguishing between the two is particularly important.

Although our review focuses on *Daphnia*, all five of these conclusions are relevant to other organisms and thus may be general organizing principles for understanding the evolutionary causes and consequences of maternal effects (e.g., Bernardo 1996; Roach and Wulff 1987; Reed and Clark 2011; Moore et al. 2019). They form the basis for future research addressing questions related to physiological and molecular mechanisms (which maternal effects mechanisms get used when; how those mechanisms interact with each other), the determinants of whether maternal effects are strong or weak, and the persistence of effects beyond first-generation offspring.

### Key issues for future research

**Fig. 3:**
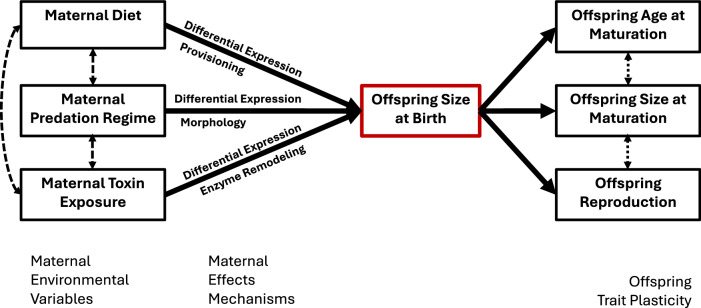
Schematic detailing the environmental attributes reviewed, the effects commonly observed, and results on offspring phenotypes. Maternal exposure is linked to changes in gene expression, provisioning, and enzymatic alterations that ultimately affect offspring egg size. Egg size and provisioning dictate key life history traits like age and size at maturation and clutch size. Dashed arrows represent potential interactions.

What proportion of maternal effects are driven by offspring size and energy provisions, other provisions, or inherited information? Many environmental characteristics induce maternal effects mediated by offspring size (mass), and a number of researchers have explored the physiological basis of energy provisions (e.g., McCauley et al. 1990; Glazier 1991; Wacker and Martin-Creuzburg 2007). Changes in offspring size at birth affect age and size at maturation, clutch size, disease susceptibility, predator-induced morphologies, resource acquisition, and other traits (Fig. 3). However, changing offspring size is not the only way mothers influence their offspring; substantial maternal effects are independent of offspring size. We lack an understanding of what proportion of maternal effects are driven by offspring size and what proportion are driven via other mechanisms or how they may interact. Such an understanding would allow us to compare maternal effects to ordinary genetic variation of size at birth and produce better predictions of evolutionary change through clear delineation of molecular constraints on evolution. Experiments could be designed to partition maternal effects between offspring size and non-size influences on standardized measures of fitness. These experiments could involve precise measurements of what maternally derived elements an embryo contains, which are then analyzed through regression techniques familiar to ecologists seeking to understand the influence of multiple factors on phenomena of interest. Alternatively, manipulative experiments that alter an embryo’s starting point (e.g., via RNAi, CRSIPR-Cas microinjection, or methylation disruption via DNMT knockout or methotrexate) could draw on the experience of molecular biology and cellular physiology. Both approaches could then be extended by using multiple clones that differ genetically in size at birth or even in genotype-environment interactions of size at birth.

Do different maternal effects driven by different aspects of the environment occur through shared physiological and molecular mechanisms? For instance, physiological responses to infochemicals released by *Daphnia* in high densities resemble those of ambient food limitation (Boersma et al. 1999), even when the media used is enriched with algae to compensate for consumption by intraspecific competitors (e.g., Seitz 1984). This suggests that the molecular pathways behind the physiological changes start from different sensory mechanisms and potentially feed into shared components, yielding physiological similarities.

Is the greater manifestation of maternal effects when the offspring environment is poor simply a byproduct arising from stressed offspring that are less able to optimize their own lives, or is it favored by selection? This is a difficult question to answer in lab environments that are kept constant over time or shifted between generations to provoke potential plastic responses. As a model system, *Daphnia* offer an extensive history of fieldwork that allow the scale and timing of environmental variation to be matched to the conditions in which a population has evolved. Furthermore, experiments can be conducted in the field, and use natural changes to assess the conditions under which strong maternal effects are advantageous.

To what extent do ancestral effects persist beyond the first generation of offspring? This is a significant question for maternal effects broadly, but like research with other taxa, almost all *Daphnia* studies focus exclusively on first generation offspring. Our knowledge of seasonal variation in lakes and ponds allow framing hypotheses about effects that may persist across more generations. Given the cyclically parthenogenetic nature of most *Daphnia*, such multigenerational studies may need to address offspring sex ratio, investment in dormancy, and male performance. It is also an open question of whether maternal effects in asexually produced, immediately developing offspring are predictive of maternal effects that occur in conjunction with dormant, usually sexual, egg production. Energetically, these types of reproduction are very different, but that does not mean that other types of provisioning also differ.

**Fig. 4:**
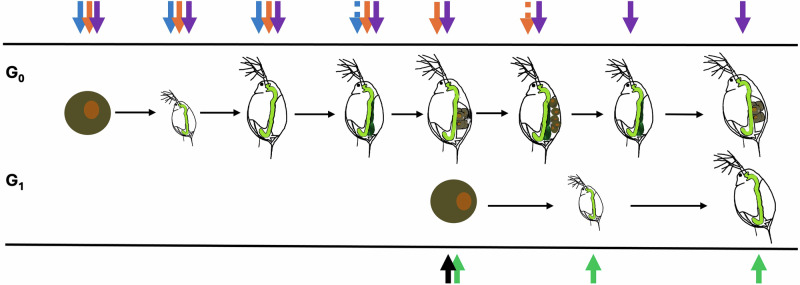
Diagram showing when a maternal effect can be expected based on the timing of exposure during development. Arrows on top refer to the maternal generation (G_0_), arrows on bottom to the embryonic/offspring generation (G_1_). Solid blue arrows indicate when the environmental exposure results in an unambiguous maternal effect for the first clutch (C_1_) of G_0_ offspring; the dotted blue arrow indicates when an unambiguous maternal effect is less likely because packaging in oogenesis has already begun. Since maternal provisioning occurs during oogenesis, maternal exposure to environmental stimuli at the dotted blue arrow is less likely to alter the phenotypic outcomes of C_1_ embryos in a meaningful way but could result in potential provisioning differences for C_2_ offspring (eggs housed in the brood chamber are not supplied with materials by the mother after extrusion). The solid black arrow indicates the point at which some aspects of the environment may directly affect the phenotype of offspring from the first eggs produced by G_0_, rendering purported maternal effects ambiguous. Solid orange and violet arrows indicate exposure times for an unambiguous maternal effect in C_2_ and C_3_, respectively. Green arrows show where exposure of the offspring generation (G_1_) can result in an unambiguous maternal effect for the first clutch produced in the subsequent generation.

From a practical perspective, embryonic exposures will always co-occur with maternal exposure in natural populations of *Daphnia*. Carefully distinguishing between their effects has not been necessary for understanding either the *causes* or *consequences* of maternal effects. However, understanding *mechanisms* of maternal effects depends on separating these exposures and considering the timing of the exposure (Fig. 4). Wade (1998) outlined three stages where maternal effects can occur, only one of which can apply to *Daphnia*. Embryos in the brood chamber have no feeding support or direct connection to the mother (Zaffagnini 1987), and there is no relationship after offspring are released. This suggests that the time in which maternal effects can occur in *Daphnia* would be prior to and during embryogenesis up to vitellogenesis (darkening of ovaries). Maternally derived provisions of phosphorus, PUFA/sterols, calcium, and transcription factors can only be allocated prior to the release of the eggs into the brood chamber. Collectively, data from the diet and toxic algae experiments suggest that offspring are indeed provisioned with these components during embryogenesis resulting in notable maternal effects.

Further possibilities come from the simple fact that eggs can be removed from the mother’s brood chamber easily and will still develop normally, allowing them to be exposed to whatever conditions an experiment requires. Predation experiments can carefully control for direct embryonic exposure by removing the mother from media containing kairomones once vitellogenesis occurs to prevent embryonic detection of the kairomone, allowing researchers to separate maternal effects from direct embryonic exposure.

These key issues will benefit from research that integrates population ecology, genetic variation, phenotypic plasticity, and molecular mechanisms. *Daphnia* is a model system with a long history of research addressing the first three components, but the study of molecular mechanisms in *Daphnia* is much newer. While some types of molecular mechanisms will involve provisioning offspring with active biomolecules, epigenetic mechanisms potentially drive substantial maternal effects, since they are responsible for directing both developmental changes in gene expression and within-generation responses to the environment.

### Epigenetics: mechanisms for maternal effects

Epigenetics can broadly be thought of as those molecular mechanisms that provide regulatory information for the execution of a genetic program. These mechanisms include, among others, DNA methylation, small RNAs, and histone modification. In some cases, that information can be inherited across cell divisions or even organismal generations in addition to information encoded by a DNA sequence. Epigenetic mechanisms may drive maternal effects by directly altering maternal actions such as provisioning or through non-genetic inheritance that alters gene expression and development in the offspring.

*Daphnia* epigenetic studies have focused on DNA methylation, highlighting environmental cues that result in maternal effects that induce stable, transmissible epigenetic alterations (Vandegehuchte et al. 2009; Jeremias et al. 2018; Trijau et al. 2018; Nguyen et al. 2020; reviewed in Harris et al. 2012; Wojewodzic and Beaton 2017). *D. magna* undergo methylation alterations in response zinc and salt stress (Vandegehuchte et al. 2009; Jeremias et al. 2018), resulting in maternally derived genomic hypomethylation linked with changes in gene transcription in offspring (Vandegehuchte et al. 2009). Resource limitation likewise alters methylation across the genome (Hearn et al. 2019, 2021), though alterations vary among clones. Results from Nguyen et al. (2020) and Agrelius et al. (2023) show that genes encoding DNA methyltransferases, enzymes responsible for adding and removing methyl groups to DNA, are upregulated under low food conditions and during several embryonic stages of development. Diets rich in vitamin B_12_ result in increased reproductive performance (Keating 1985) and was linked with global hypermethylation of *Daphnia* genome (Kusari et al. 2017) capable of being transmitted across generations.

Work by Asselman et al. (2015) revealed very low global methylation percentages that respond dynamically when exposed to predator kairomones, resulting a fivefold difference across genotypes, and exposure to a toxic cyanobacterium resulted in differential gene body methylation (Asselman et al. 2017). Predator exposure induced a clear shift in methylation of more than 2000 genes between two generations in *D. ambigua* (Schield et al. 2016) that coincided with a reprogramming of life history traits for future generations. Neurosignaling pathways used by *Daphnia* to induce morphological defenses and alter life history traits in response to kairomones (Weiss et al. 2012, Weiss et al. 2015a, 2015b, 2018) are documented to be under various levels of epigenetic control in vertebrates (Shrestha and Offer 2016; Bekdash 2019), further indicating the connection between epigenetics and environmental stimuli.

## Prospectus

*Daphnia* are poised to be the model system that allows researchers to integrate a history of ecological research with a future that investigates epigenetic mechanisms, providing a holistic understanding of the ecology and evolution of maternal effects. Adaptive predictions can be grounded in our strong understanding of the environmental context of *Daphnia* population dynamics. The combination of sexual and asexual reproduction in the *Daphnia* life cycle provides genetic variation at multiple scales, allowing experiments on genetically identical individuals with natural genomes. Functional genetic tools, genomic data, and initial forays into epigenetics permit linking organismal phenotypic responses to molecular mechanisms. Altogether, the mysteries of how and why non-genetic information gets passed to future generations can be unraveled in these small crustaceans.

## Supplementary information

Supplemental Table
