# Correction: Maternal effects in the model system *Daphnia*: the ecological past meets the epigenetic future

**DOI:** 10.1038/s41437-025-00771-z

**Published:** 2025-05-23

**Authors:** Trenton C. Agrelius, Jeffry L. Dudycha

**Affiliations:** 1 Department of Biological Sciences, University of Notre Dame, Notre Dame, IN USA; 2 Department of Biological Sciences, University of South Carolina, Columbia, SC USA

**Keywords:** Epigenetics, Evolution, Ecology

Correction to: *Heredity* 10.1038/s41437-024-00742-w, published online 08 January 2025

In this article Fig. 3 was given erroneously. This has been corrected.
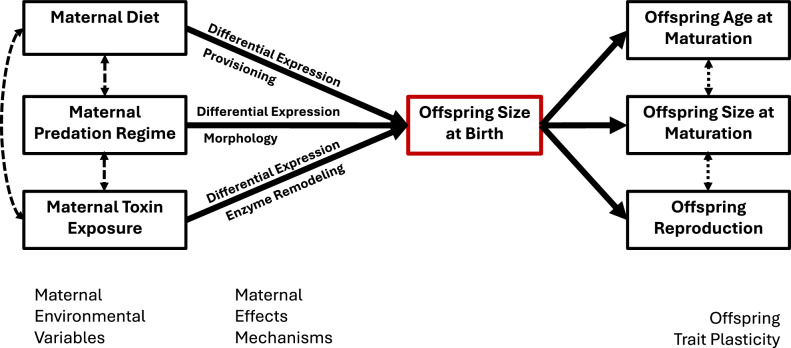


The original article has been corrected.

